# Depletion of *Toxoplasma* adenine nucleotide translocator leads to defects in mitochondrial morphology

**DOI:** 10.1186/s13071-022-05295-7

**Published:** 2022-05-31

**Authors:** Yihan Wu, Zhu Ying, Jing Liu, Zhepeng Sun, Shuang Li, Qun Liu

**Affiliations:** 1 National Animal Protozoa Laboratory, College of Veterinary Medicine, China Agricultural University, No. 2 Yuanmingyuan West Road, Beijing, 100193 China; 2 Key Laboratory of Animal Epidemiology of the Ministry of Agriculture, College of Veterinary Medicine, China Agricultural University, Beijing, China

**Keywords:** *Toxoplasma gondii*, Adenine nucleotide translocase, Mitochondria

## Abstract

**Background:**

Adenine nucleotide translocase (ANT) is a protein that catalyzes the exchange of ADP/ATP across the inner mitochondrial membrane. Beyond this, ANT is closely associated with cell death pathways and mitochondrial dysfunction. It is a potential therapeutic target for many diseases. The function of the ANT in *Toxoplasma gondii* is poorly understood.

**Methods:**

The CRISPR/CAS9 gene editing tool was used to identify and study the function of the ANT protein in *T. gondii*. We constructed* T. gondii* ANT transgenic parasite lines, including endogenous tag strain, knockout strain and gene complement strain, to clarify the function and location of TgANT. Mitochondrial morphology was observed by immunofluorescence and transmission electron microscopy.

**Results:**

*Toxoplasma gondii* was found to encode an ANT protein, which was designated TgANT. TgANT localized to the inner mitochondrial membrane. The proliferation of the Δ*ant* strain was significantly reduced. More important, depletion of TgANT resulted in significant changes in the morphology and ultrastructure of mitochondria, abnormal apicoplast division and abnormal cytoskeletal daughter budding. In addition, the pathogenicity of the Δ*ant* strain to mice was significantly reduced.

**Conclusions:**

Altogether, we identified and characterized the ANT protein of *T. gondii*. Depletion of TgANT inhibited parasite growth and impaired apicoplast and mitochondrial biogenesis, as well as abnormal parasite division, suggesting TgANT is important for parasite growth.

**Supplementary Information:**

The online version contains supplementary material available at 10.1186/s13071-022-05295-7.

## Background

*Toxoplasma gondii* is an obligate intracellular parasite known to infect humans and all other warm-blooded animals [1]. Being apicomplexan, *T. gondii* presents a single, ramified mitochondrion [2]. Compared to the genes found in the mammalian mitochondrial genome, the *Toxoplasma* mitochondrial genome is severely reduced, encoding only three proteins, cytochrome* b*, cytochrome* c* oxidase I and cytochrome* c* oxidase III, along with interspersed ribosomal RNA elements [3–5]. Due to the mitochondrion being essential for survival of the parasite at all life-cycle stages and, more importantly, its genetic and structural divergence from the host mitochondria, it is not surprising that the parasite mitochondrion is considered to be an ideal drug target.

In mammals, adenine nucleotide translocase (ANT) is the most abundant protein in mitochondria, accounting for 10% of the total mitochondrial protein content, and plays an important role in the transport of metabolites and cofactors across the inner mitochondrial membrane (IMM) [6–8]. In addition, ANT appears to be a component of the mitochondrial permeability transition pore (mPTP), whose opening induces the release of multiple mitochondrial intermembrane proteins, including various activators of the intrinsic pathway of apoptosis, into the cytoplasm, which then leads to cell death. Helena and colleagues found that nitric oxide (NO), peroxynitrite, and 4-hydroxynonenal could directly act on ANT to induce mPTP opening and apoptosis [9]. The recent report showed that ANT was also closely related to maintenance of the normal structural and functional of mitochondria. In *Saccharomyces cerevisiae*, it has been shown that the mutation of ANT induced structural and functional changes of mitochondria [10]. In cancer, ANT has been identified as a family of proteins involved in mitochondrial dysfunction [11, 12]. These studies indicate that ANT is closely associated with mitochondrial dysfunction and that it is clearly an important target for drug development. The function of ANT proteins in *T. gondii* is poorly understood.

In this study, we showed that *T. gondii* encodes an ANT protein, which we named TgANT. To investigate the physiological role of TgANT, we constructed *ant* gene knockout strain (Δ*ant*) and demonstrated that TgANT was important for maintaining mitochondrial morphology. Also, its depletion significantly inhibited the proliferative capacity of *T. gondii* in vitro and pathogenicity to mice.

## Methods

### Parasites and host cells

Human foreskin fibroblasts (HFFs) and Vero cells (African green monkey kidney cells) were obtained from the Cell Bank of the Chinese Academy of Sciences (Shanghai, China). RHΔ*ku80* was used as the parental strain to construct the gene knockout strain. Parasites were maintained in vitro on Vero cells or HFFs in DMEM (M&C, Beijing, China) supplemented with penicillin/streptomycin with 2% fetal bovine serum (FBS; Gibco, Thermo Fisher Scientific, Waltham, MA, USA) at 37 °C and 10% CO_2_. The medium was changed 12 h after inoculation.

### Plaque assays

The plaque experiment was performed using HFFs cultured in 12-well cell culture plates. When HFFs grew to 80%–90% confluency, the medium was replaced with fresh medium containing 2% FBS. Freshly egressed parasites were purified by passage through a 5.0 µm-filter and collected from the cells for counting; approximately 150 tachyzoites were inoculated per well and incubated at a constant temperature in an incubator at 37 °C with 5% CO_2_ for 6–7 days. The medium was then removed and the cells washed twice with phosphate buffered saline (PBS). They were then fixed with 4% paraformaldehyde for 30 min and stained in crystal violet solution for 1 h. The plaque area was determined by pixel counting using Photoshop C6S software (Adobe Inc. San Jose, CA, USA); data were compiled from three independent experiments.

### Construction of transgenic strains

All primers and plasmids used in this study are listed in Additional file 1: Table S1.

To construct the Δ*ant* strain, we first constructed the pTCR-TgANT-CD plasmid for homologous recombination and the pSAG1-CAS9-U6gRNA plasmid for gene disruption. We amplified the 5′ flank and 3′ flank of TgANT from the genomic DNA of RHΔ*ku80* strain. Chloramphenicol (CAT) was fused with red fluorescent protein (RFP) to form the CAT-RFP cassette, which was used for positive screening, and this cassette was cloned from the pTCR-CD plasmid preserved in our laboratory. The corresponding fragments were then used to construct the plasmid pTCR-TgANT-CD by seamless cloning. The pTCR-TgANT-CD plasmid and the corresponding CRISPR–Cas9 plasmid were linearized by PCR and transfected into RHΔ*ku80* strain, and the homolog recombination sequences were identified by PCR.

To generate the TgANT-FLAG parasite line, we considered a fragment upstream of the translation stop codon (approx. size: 42 bp) as the 5′ flank homologous arm and a fragment (approx. size: 42 bp) downstream of the genomic RNA as 3′ flank. The CAT cassette used for positive screening and the vector backbone were amplified from the pLIC-3 × FLAG-CAT plasmid. The pLIC-TgANT-3 × FLAG-CAT plasmid and the corresponding CRISPR–Cas9 plasmid were linearized by PCR and transfected into RHΔ*ku80* strain.

To generate the TgANT-FLAG-TOM40-HA parasite line, 59-bp PCR primers containing the 42-bp fragments upstream of the TOM40 protein translation stop codon and downstream of the Cas9 break site were designed to amplify the PCR products obtained from a plasmid-containing pLIC-3 × HA-DHFR. Purified PCR products of the HA-DHFR cassette were co-transfected with the CRISPR/Cas9 plasmid into TgANT-FLAG parasites and selected with DHFR after electroporation.

To generate the gene complement (iΔ*ant*) strain, the *uprt* gene was disrupted by the *uprt*-specific CRISPR-Cas9 plasmid and the complete open reading frame of TgANT was amplified from the complementary DNA (cDNA) of the parasite and inserted into p5′UPRT-HA-DHFR-3′UPRT. PCR-amplified fragments were transfected with *uprt*-specific CRISPR-Cas9 plasmid into the Δ*ant* strain. Negative selection was performed using floxuridine (FUDR).

### Immunoblotting and immunofluorescence assays

Immunofluorescence assays (IFAs) were performed as described previously [13]. Briefly, tachyzoite-infected HFFs were fixed with 4% paraformaldehyde. After three washes with PBS, the cell membranes were permeabilized and samples were blocked by incubation with 0.25% Triton X-100/PBS and 3% bovine serum albumin for 30 min at room temperature. The primary antibodies used were rabbit or mouse anti-FLAG (1:50; Sigma-Aldrich, St. Louis, MO, USA), rabbit or mouse anti-HA (1:50; Sigma-Aldrich) and rabbit anti-GAP45 (1:200). 4′,6-Diamidino-2-phenylindole (DAPI; 1:100; Sigma-Aldrich) and secondary antibodies (FITC-conjugated goat anti-mouse IgG [H+L], 1:50; Cy3-conjugated goat anti-rabbit IgG [H+L], 1:100) were incubated together. Images were obtained using a Leica confocal microscope system (TCS SP52; Leica Microsystems, Wetzlar, Germany).

For the immunoblotting assays, 1 × 10^7^ parasites were collected and purified by filtration through a 5-µm filter membrane and lysed with RIPA buffer (Huaxinbio, Beijing, China). The primary antibodies used were rabbit or mouse anti-FLAG (1:500; Sigma-Aldrich), rabbit or mouse anti-HA (1:500; Sigma-Aldrich), mouse anti-actin (1:5000). Horseradish peroxidase-conjugated antibodies were used as secondary antibodies (1:5000; Invitrogen, Thermo Fisher Scientific).

### Invasion assay

The invasion assay was carried out as previously described [14]. Briefly, parasite invasion was assessed using an invasion assay whereby 1 × 10^7^ artificially egressed parasites were inoculated on HFFs growing in 12-well plates, incubated for 30 min at 37 °C and then washed with PBS. Cells were then fixed with 4% formaldehyde and stained with rabbit anti-SAG1 polyclonal antibody (1:200) and DAPI. The percentage of invasion was represented as numbers of vacuoles per host cell. For each experiment, a minimum of 100 parasites were counted from 10 fields per treatment. Three independent experiments were performed.

### Intracellular replication assay

Human foreskin fibroblasts growing in 12-well plates seeded on coverslips were inoculated with 1 × 10^5^ parasites and cultured for 30 min. The cells were then washed 3 times with PBS; after the final wash, the solution was discarded, fresh medium was added and the cells were then continuously cultured for 20 h. All parasites were visualized by an IFA using a rabbit anti-GAP45 polyclonal antibody (prepared by our laboratory). Cells were analyzed by fluorescence microscopy. The number of parasites per vacuole was determined by counting at least 100 vacuoles. The experiment was repeated three separate times.

### Pathogenicity assay

BALB/c mice (5 mice per strain) were infected with 100 parasites, and survival was observed for > 3 weeks. Three independent experiments were performed.

### Statistical analysis

Data were analyzed using GraphPad Prism 9.0 (GraphPad Inc., San Diego, CA, USA). All data were analyzed with the two-tailed Student's t-test. Statistical analysis of survival curves was analyzed by “curve comparison.”

## Results

### TgANT protein is expressed in *T. gondii*

**Fig. 1:**
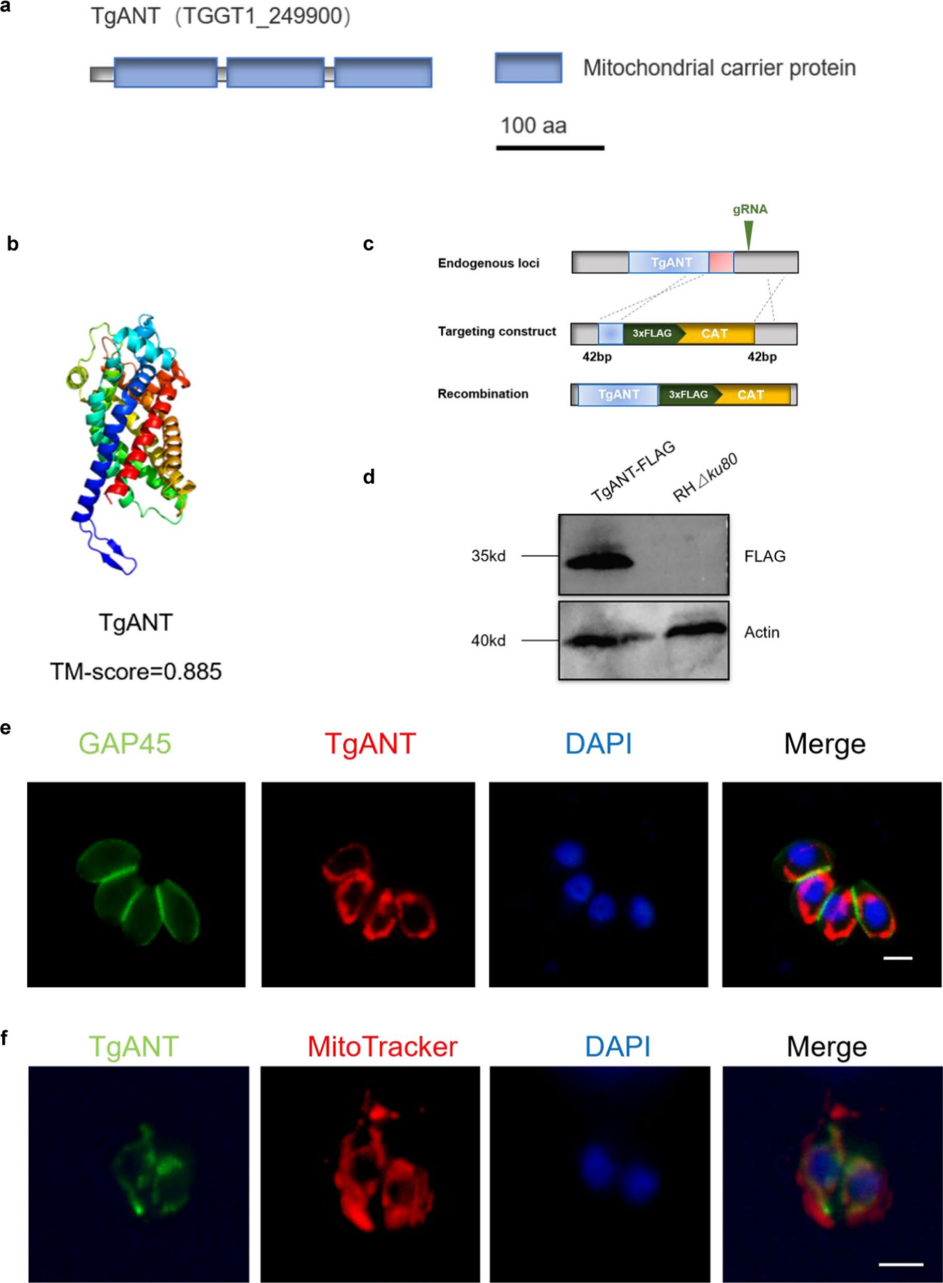
Identification and localization of TgANT protein. **a** Domain architecture of TgANT in *Toxoplasma gondii*. The domains were predicted by databases of protein families (Pfam). **b** Three-dimensional (3D) structures of TgANT predicted by Ab Initio Modeling using trRosetta. The quality of trRosetta modeling results is mainly based on its TM-score, which is between 0 and 1. The closer to 1, the higher the accuracy of the modeling and the greater the credibility. **c** Strategy for the construction of the strain with the FLAG endogenous epitope tag. **d** Western blot confirmed the expression of FLAG-tagged TgANT in parasites. Actin was used as a control. **e** IFA assays indicated that TgANT showedthe typical lasso-shaped mitochondria-like localization in intracellular tachyzoites. Parasites were labeled with rabbit anti-GAP45 (green), mouse anti-FLAG (red) and DAPI (blue). Scale bar, 2 μm. **f** IFA results showed the colocalization of anti-TgANT signal (green) and MitoTracker (red). MitoTracker was used as a mitochondrial marker. Scale bar: 2 μm. Abbreviations: CAT, chloramphenicol; DAPI,4′,6-Diamidino-2-phenylindole; IFA, immunofluorescence assay; TgANT, adenine nucleotide translocase in* T. gondii*

A bioinformatics analysis was performed to assess the conservation of known ANT proteins from yeast and human in the genome of the parasite. We identified a putative ANT protein (TGGT1_249900) in the ToxoDB database (ToxoDB), which we named TgANT. The prediction of Pfam database (Pfam: Search Pfam [xfam.org])showed that TgANT contained three mitochondrial carrier protein domains (Fig. 1a). The modeling results of trRosetta (https://robetta.bakerlab.org) showed that TgANT was similar and closely related to *Saccharomyces cerevisiae* ANT (PDB ID: 4C9H), and the confidence of the predicted model was very high (with the estimated template modeling score [TM-score] of TgANT = 0.885) (Fig. 1b). We performed phylogenetic analyses to determine the evolutionary relationships of TgANT using MEGA7 software from *T. gondii*, *S. cerevisiae, Plasmodium falciparum*, *Cyclospora cayetanensis, Arabidopsis thaliana*, *Cardiosporidium cionae*, *Homo sapiens* and other species. We found that TgANT was similar and closely related to *C. cayetanensis* and *C. cionae*, which are on the same branch (Additional file 2: Fig. S1a).

To identify the localization of TgANT, we introduced a FLAG tag at the C-terminus of TgANT in the RHΔ*ku80* strain (Fig. 1c). Western blot verified the expected molecular mass of approximately 35 kDa for TgANT-FLAG (Fig. 1d). Using endogenously tagged ANT, we found that ANT was localized to the mitochondrion in *T. gondii*, as demonstrated by colocalization with the mitochondrial marker MitoTracker (Fig. 1e, f).

### TgANT localizes to the IMM

**Fig. 2:**
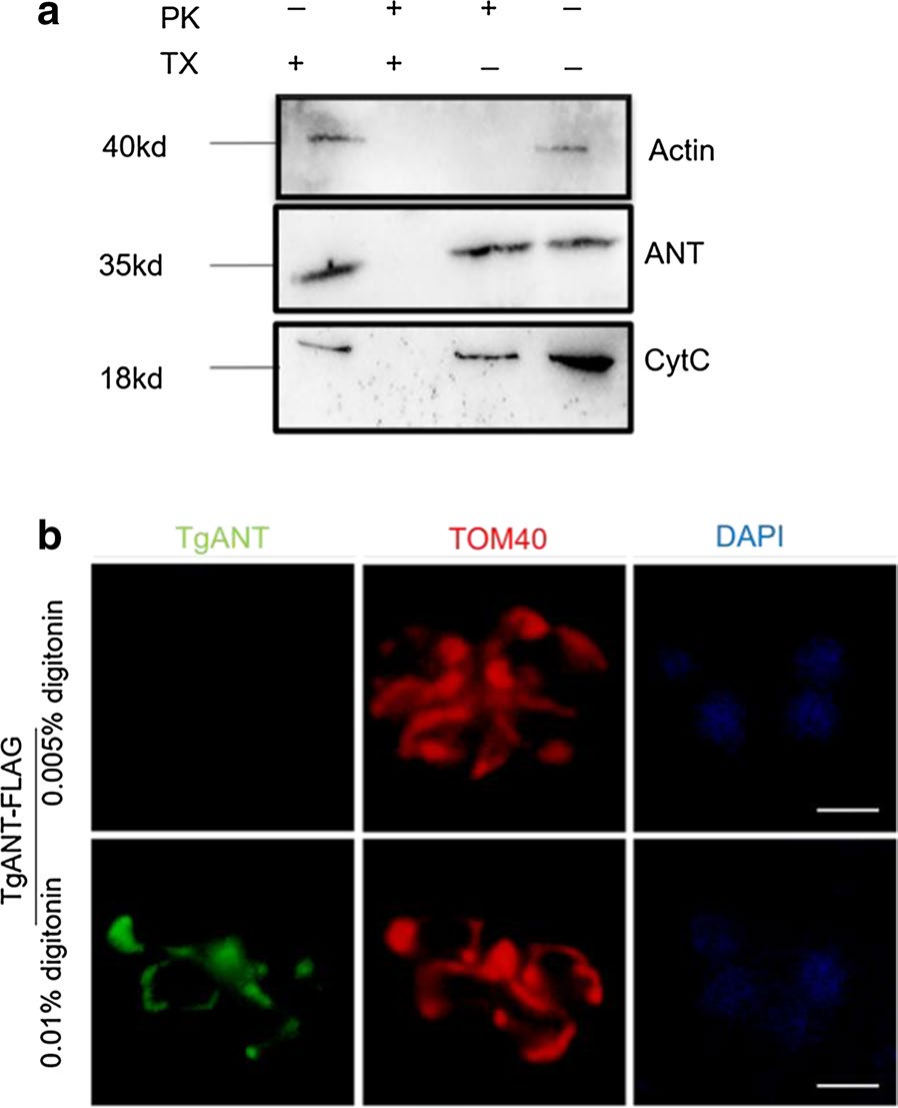
TgANT localizes to the IMM. **a** The PK protection assay was used to determine the accessibility of TgANT to 0.1 mg/ml PK in the presence or absence of the detergent 0.5% TX. After permeabilization by TX, PK could easily pass through organelle membranes to the stroma and digest stroma proteins nonspecifically. Actin was used as a cytoplasmic protein marker, and PK did not need the assistance of TX permeabilization solution for its nonspecific degradation process. CytC is a marker of mitochondrial intermembrane space, and its degradation by PK requires the synergistic effect of the TX permeabilizer. **b** The TgANT-FLAG-TOM40-HA strain is permeabilized with 0.005% or 0.01% digitonin. TOM40 was used as the markers of the mitochondrial outer membrane. Using 0.005% digitonin only allowed the detection of TOM40, while using 0.01% digitonin, both TOM40 and TgANT were detected, suggesting that TgANT is associated with the IMM. Parasites were labeled with mouse anti-FLAG (green), rabbit anti-HA (red) and DAPI (blue). Scale bar: 2 μm. Abbreviations: CytC, cytochrome* c*; IMM, inner mitochondrial membrane; PK, proteinase K; TX, Triton-X-100

In mammals, ANTs are located on the IMM [15]. The prediction result of the TMHMM Server v.2.0 (TMHMM—2.0—Services—DTU Health Tech) showed that TgANT contained four transmembrane domains (Additional file 2: Fig S1b). To determine whether TgANT is associated with the inside of the mitochondria, we completed a proteinase K (PK) protection assay with cytochrome* c *(CytC) and actin as controls. CytC was located in the intermembrane space of mitochondria and was fully protected from PK digestion without Triton X-100 permeabilization, whereas actin was digested by PK even without the addition of detergent. TgANT, like CytC, had to be treated with detergent to be digested by PK, indicating that TgANT was likely to be associated with the IMM (Fig. 2a). To confirm the accuracy of the PK protection assay results, we also performed IFA assays after permeabilization with various concentrations of digitonin, using the detection of TOM40 (a marker of the outer mitochondrial membrane) to monitor mitochondrial permeabilization [16]. The results showed that TOM40, but not TgANT, could be detected using 0.005% digitonin, but that TgANT could be detected when the concentration of digitonin was increased (0.01%). These results suggest that TgANT localizes to the IMM (Fig. 2b).

### Deletion of TgANT inhibits *Toxoplasma* growth

**Fig. 3:**
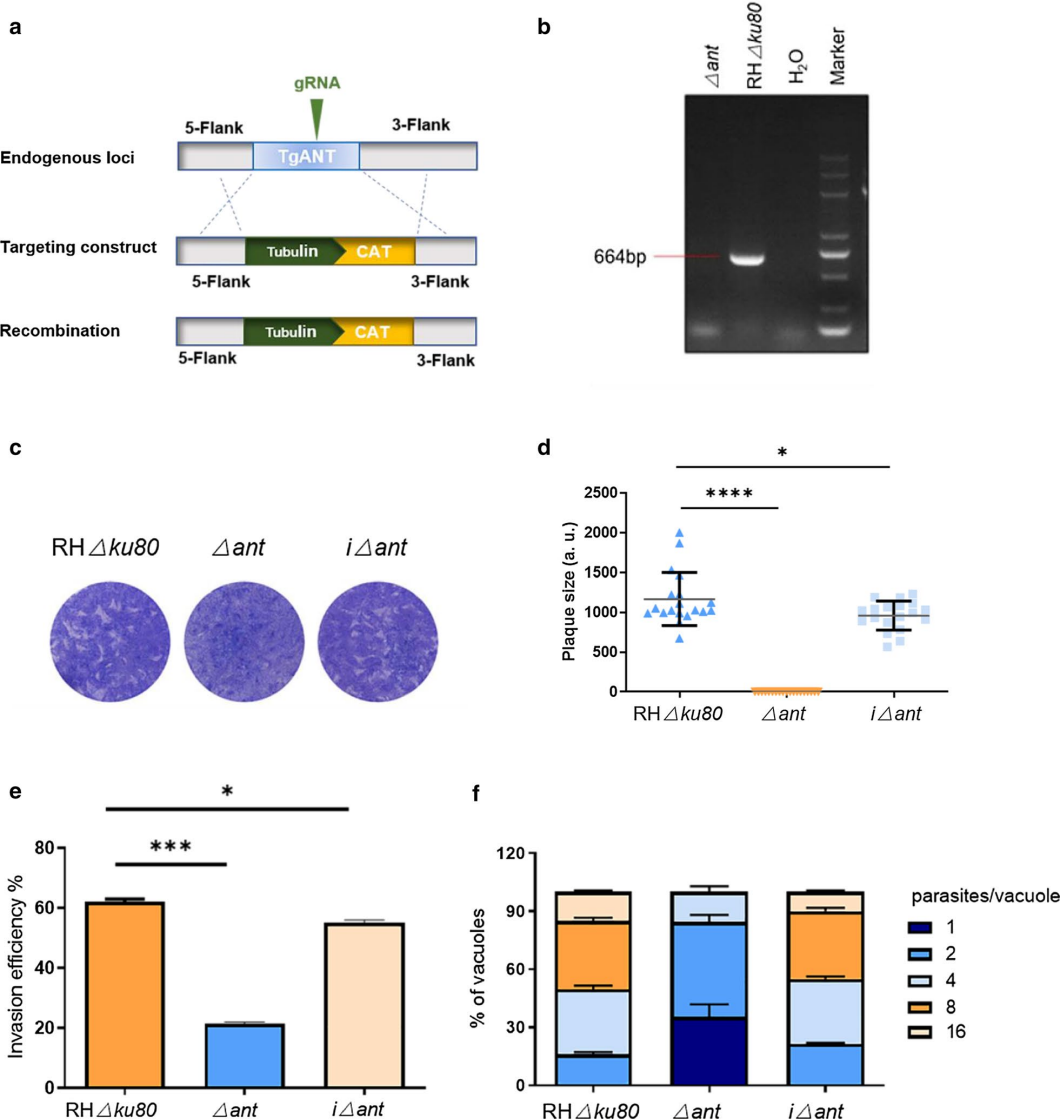
Lack of TgANT decreased the growth of parasites. **a** Schematic of CRISPR/Cas9 strategy used for gene deletion. **b** PCR analysis confirmed knockout of the *ant* gene in the gene deletion mutant (Δ*ant*). **c** Plaque assays were used to compare the overall growth ability of the RHΔ*ku80*, Δ*ant* and iΔ*ant* strains. Each well was infected with 150 parasites, and plaques were stained after 7 days. **d** Plaque areas were measured by counting pixel points in Photoshop C6S software (Adobe Inc.). The data are compiled from three independent experiments. The plaque assay showed that *Δant* strains had distinct growth defects. Asterisks indicate significant differences at *P < 0.05 and ****P < 0.0001. **e** Parasite invasion into HFFs was examined. The invasion rate of the Δ*ant* strain was 51%, which was significantly different from that of the RHΔku80 strain (62%). Data are presented as the mean ± standard deviation of the results from three assays. Asterisks indicate significant differences at*P < 0.05 and ***P < 0.001. **f** Comparison of intracellular replication ability of Δ*ant*, iΔ*ant*, and RHΔ*ku80* strains. In the Δ*ant* strain, the proportions of PVs containing 1, 2 and 4 parasites was 35, 49 and 51%, respectively, and it was almost impossible to observe PVs containing > 4 parasites. The proportions of PVs containing 1, 2, 4, 8 and 16 parasites in the RHΔku80 strain was 0, 1%, 34, 35 and 15%, respectively. HFFs, Human foreskin fibroblasts; PVs, parasitophorous vacuoles; Δ, gene knockout; iΔ, gene complement

To further investigate the function of TgANT, we used CRISPR/CAS9 technology to construct the gene deletion mutant (Δ*ant*) by double homologous recombination in the RHΔ*ku80* strain (Fig. 3a). PCR analysis confirmed knockout of the target gene (Fig. 3b). To assess the viability of Δ*ant*, we monitored the formation of plaques by continuous 7-day culture. A significant reduction in plaque formation area was observed in the Δ*ant* strain, with the plaque of the Δ*ant* strain barely visible by eye after 7 days (Fig. 3c–d). The results of the invasion assay also revealed that TgANT was essential for parasite survival, with significantly impaired host cell invasion in Δ*ant* as compared to RHΔ*ku80* (Fig. 3e). Intracellular replication was also significantly reduced in Δ*ant* (Fig. 3f). These results are consistent with the phenotypic values marked by ToxoDB database (TgANT phenotypic value: − 3.78). To ensure the credibility of our results, we constructed the *ant* gene complementary strain, which was identified by PCR and IFA (Additional file 3: Fig. S2). Complementation of the *ant* gene rescued the invasion and intracellular replication ability (Fig. 3c–f).

### Loss of TgANT results in aberrant mitochondrial morphology

**Fig. 4:**
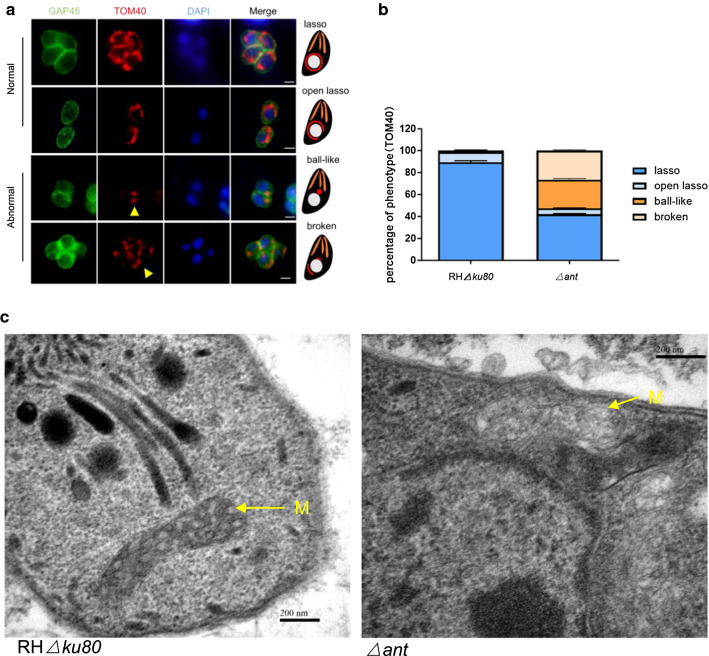
Depletion of TgANT leads to disruption of mitochondrial morphology. **a** HA endogenous tags were added to the C terminal of the mitochondrial outer membrane protein TOM40 of the Δ*ant* and RHΔ*ku80* strains. Mitochondrial outer membrane morphology was visualized by IFA. Two different mitochondrial phenotypes were identified in Δ*ant*, which we named ball-like and broken (yellow arrowhead). GAP45 (green) was used as a marker of the parasite membrane; TOM40 (red) was used as a marker of the parasite mitochondrial outer membrane; DAPI (blue) was used to stain nuclei. Mitochondrial morphology of intracellular parasites was scored as indicated. Scale bar: 2 μm. **b** Randomly selected vacuoles (*n* = 100) from two independent experiments were quantified. No significant changes were seen in the RHΔ*ku80* strain, but the mitochondrion of the *ant* gene knockout parasites showed severe morphological defects, classified as “broken” (prevalence: appox. 27%) and “ball-like” (prevalence: approx. 26%). **c** Representative transmission electron microscopy images of Δ*ant* and RHΔ*ku80* parasites. The deletion of *ant* resulted in a loss of density and a reduced number of mitochondrial cristae. The mitochondria of RHΔ*ku80* parasites had a regular morphology with a clear cristae structure. M, Mitochondrion. Scale bars: 200 nm

In *S. cerevisiae*, mutation of ANT was found to induce changes in mitochondrial morphology [17]. We hypothesized that TgANT depletion would lead to changes in mitochondrial morphology. Thus, we introduced a C-terminal HA epitope into TOM40 in the Δ*ant* and RHΔ*ku80* strains and then observed the mitochondrial morphology of the two strains using the IFA. In the RHΔ*ku80* strain, 97% of the mitochondria showed the typical lasso-shaped or open lasso shape morphology, as previously reported [18] (Fig. 4a,b). In contrast, we identified two different mitochondrial phenotypes in Δ*ant*, which we named ball-like (approx. 26%) and broken (approx. 27%) (Fig. 4a, b).

The morphology of the mitochondrion was examined in more detail using transmission electron microscopy. In Δ*ant*, mitochondria appeared to have lost density and a reduced number of mitochondrial cristae were observed (Fig. 4c). On the contrary, the RHΔ*ku80* straine presented well-organized organelles (Fig. 4c). These results indicate that TgANT is very important for maintaining the normal structure of mitochondria.

### Deletion of TgANT results in asynchronous replication

**Fig. 5:**
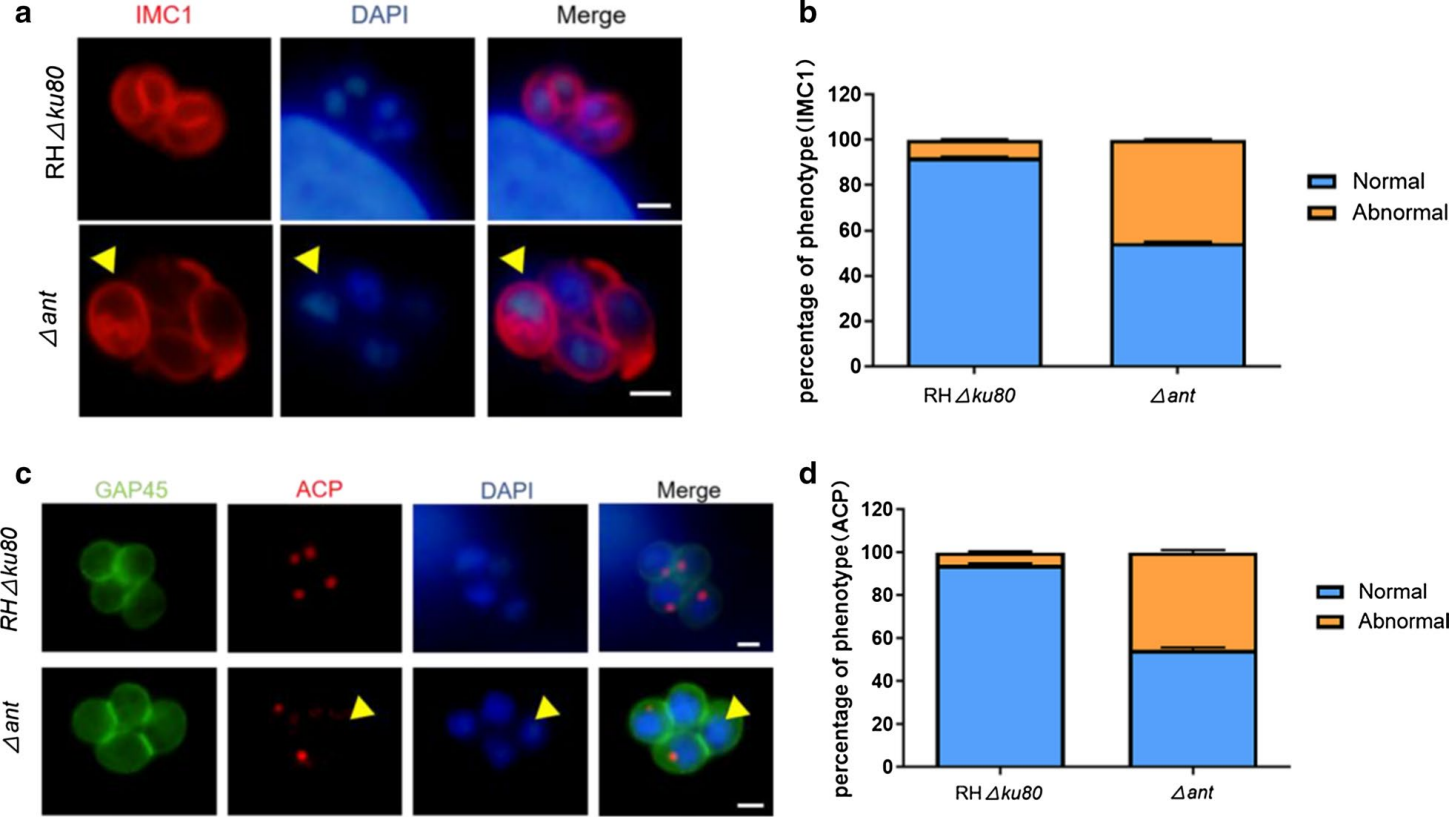
Loss of TgANT results in asynchronous replication. **a** IMC1 (red), the marker of different stages of cell division; DAPI (blue) was used to stain nuclei. Arrowheads indicate parasites with abnormal cytoskeletal daughter budding. Scale bar: 2 μm. **b** Rate of asynchronous PVs in Δ*ant* (approx. 45%) and RHΔ*ku80* (approx. 7%). Randomly selected vacuoles (*n* = 100) from two independent experiments were quantified. **c** Apicoplast of *Δant* and RHΔ*ku80* strains was determined by immunofluorescence. ACP was used to mark the apicoplast. Arrows indicate parasites with abnormal apicoplast division. Scale bar: 2 μm. **d** Loss rate of the apicoplast in Δ*ant* (appox. 44%) and RHΔ*ku80* (approx. 7%). Randomly selected vacuoles (*n* = 100) from two independent experiments were quantified. Abbreviations: ACP, Acyl carrier protein; IMC1, Inner membrane complex protein 1

Results from several studies have implied that proper mitochondrial morphology is a prerequisite for mitochondrial division in *T. gondii* and that mitochondrial division is tightly linked to cell division [19–21]. To determine whether the change in mitochondrial morphology in Δ*ant* would contribute to abnormal cell division, we investigated the cell division of the *ant* gene knockout strain. The inner membrane complex protein 1 (IMC1) had been used in previous studies to examine daughter scaffold formation in *T. gondii* [22, 23]. In the present study, we used IMC1 as a marker for indirect immunofluorescence to observe the daughter cell budding of parasites. The results showed that compared to RHΔ*ku80*, daughter cell budding was asynchronous in some parasitophorous vacuoles (PVs)in Δ*ant* (approx. 45%), suggesting that deletion of the TgANT led to abnormal cell division (Fig. 5a, b). The apicoplast is a metabolically relevant secondary endosymbiotic organelle whose segregation occurs concurrently with nuclear division and daughter cell budding [23]. We found that the apicoplast was lost in approximately 42% of the vacuoles after deletion of TgANT (Fig. 5c, d).

### The loss of *ant* significantly reduced the pathogenicity of *T. gondii* in mice

**Fig. 6:**
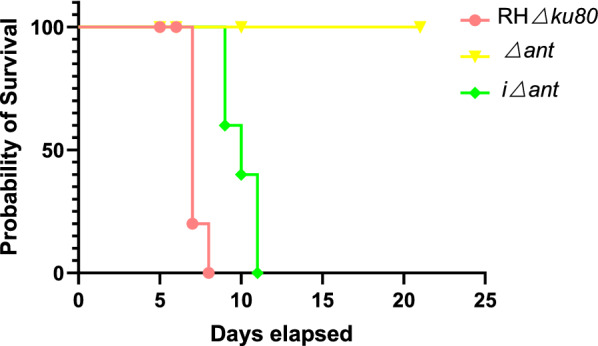
TgANT knockout parasites show significantly reduced pathogenicity to mice. Five BALB/c mice were infected with 100 RHΔ*ku80*, Δ*ant* or iΔ*ant* tachyzoites, and their survival was assessed over 21 days

We intraperitoneally inoculated 100 RHΔ*ku80* and Δ*ant* tachyzoites into BALB/c mince to evaluate their virulence in these animals (5 mice per strain). The survival curve showed that all mice infected with 100 RHΔ*ku80* tachyzoites died within 7 days, while mice receiving 100 Δ*ant* tachyzoites survived for 21 days. Importantly, the complementation of TgANT rescued the pathogenicity to mice (Fig. 6).

## Discussion

We describe here a *T. gondii* homolog for the ANT, which we named TgANT. ANT is the most abundant protein in mitochondria, accounting for 10% of the total mitochondrial protein content [17]. In addition to catalyzing ADP/ATP exchange across the inner membrane, ANT is closely related to the cell death pathway and mitochondrial dysfunction [24]. In the present study, we found that in *T. gondii*, depletion of ANT resulted in significant growth inhibition. We also observed dramatic changes in mitochondrial morphology, in concert with abnormal cytoskeletal daughter budding, suggesting that TgANT is important for parasite growth.

In mammals, ANT has been reported to be involved in regulation of the cell death pathway and to be a critical target of apoptosis induction by NO [9]. The inhibitory effect of NO on *T. gondii* has been reported previously [25, 26]. We attempted to investigate whether the Δ*ant* strain was resistant to NO inhibition. However, proliferation of the Δ*ant* strain was severely impaired, resulting in growth defects and, therefore, we could not use the TgANT complete knockout strain for subsequent studies. We thus attempted to construct a conditional knockout strain by the auxin-inducible degron (AID) system [27], but because TgANT was a membrane protein, we did not succeed. Further studies are required to determine the relationship between TgANT and the cell death pathway.

In *S. cerevisiae*, the mutation of ANT results in disruption of mitochondrial morphology [17]. In *T. gondii*, depletion of TgANT led to defects in mitochondrial morphology. To further explore the ultrastructure of mitochondria in the Δ*ant* strain, we used transmission electron microscopy. The results showed that depletion of TgANT resulted in loss of density and a reduced number of mitochondrial cristae. However, we could not determine whether the morphological changes of mitochondria in the Δ*ant* strain was an indirect effect or merely due to *ant* gene deletion. Further studies are required to determine how TgANT affects mitochondrial morphology.

In addition to the mitochondrion, *T. gondii* also harbors another endosymbiotic organelle, the apicoplast, which is closely related to the mitochondrion [28]. We suspect that the deletion of the TgANT results in abnormal mitochondrial morphological structure, which in turn may have impaired apicoplast biogenesis. We performed indirect immunofluorescence experiments using acyl carrier protein as a marker to observe the segregation of the apicoplast. The results showed that depletion of TgANT impaired apicoplast biogenesis. Several studies have implied that proper mitochondrial morphology is tightly linked to cell division [19, 20]. We found that depletion of TgANT resulted in abnormal cytoskeletal daughter. The above results prove that TgANT is very important to the maintenance of the normal life activities of *Toxoplasma gondii*.

## Conclusion

In conclusion, we found that *T. gondii* encodes an ANT protein (TgANT), which localizes to the IMM. TgANT is crucial for the proliferation of *T. gondii* and is required to maintain the morphology of mitochondria. Depletion of TgANT impaired apicoplast and mitochondrial biogenesis, as well as abnormal parasite division.

## Supplementary Information

**Additional file 1: Table S1.** Primers used for this study.

**Additional file 2: Figure S1.** **a** protein sequence alignment of TgANT using MEGA7 from *Toxoplasma gondii*, *Saccharomyces cerevisiae, Plasmodium falciparum*, *Cyclospora cayetanensis, Arabidopsis thaliana*, *Cardiosporidium cionae*, *Homo sapiens* and other species. **b** Prediction results of TMHMM Server v.2.0 showed that TgANT has four transmembrane domains.

**Additional file 3: Figure S2.** Identification of the iΔ*ant* gene complementation strain. **a** PCR1 and PCR2 were used to amplify the 5′ homologous recombination fragment (1890 bp) and the 3′ homologous recombination fragment (1218 bp), respectively. Lanes 2 and 5 are the experimental group; lanes 1 and 4 are the negative control group; lanes 3 and 6 are the blank control groups. **b** IFA assay. GAP45 (green) marks the outline of the parasite; red indicates the target protein TgANT; DPAI (blue) marks the nucleus. Scale bar: 2 μm.

## Data Availability

All datasets generated for this study are included in the manuscript/supplementary files.

## Abbreviations

ANT: Adenine nucleotide translocase
CAT: Chloramphenicol
IMC1: Inner membrane complex protein 1
IMM: Inner mitochondrial membrane
PK: Proteinase K
PVs: Parasitophorous vacuoles
Δ: Gene knockout
iΔ: Gene complement
