# Supplementary figures and images for: Depletion of Toxoplasma adenine nucleotide translocator leads to defects in mitochondrial morphology
Source: Parasit Vectors. 2022 May 31;15:185. doi: 10.1186/s13071-022-05295-7 (PMC9158195; doi:10.1186/s13071-022-05295-7)

Figure S1

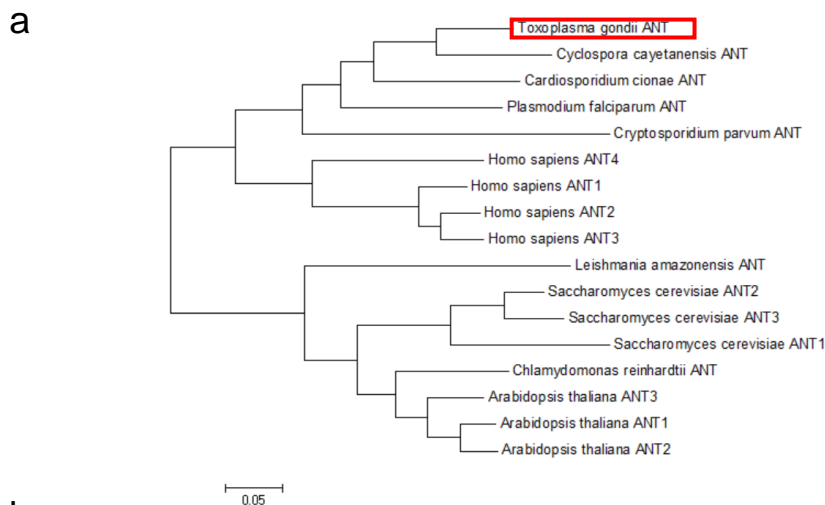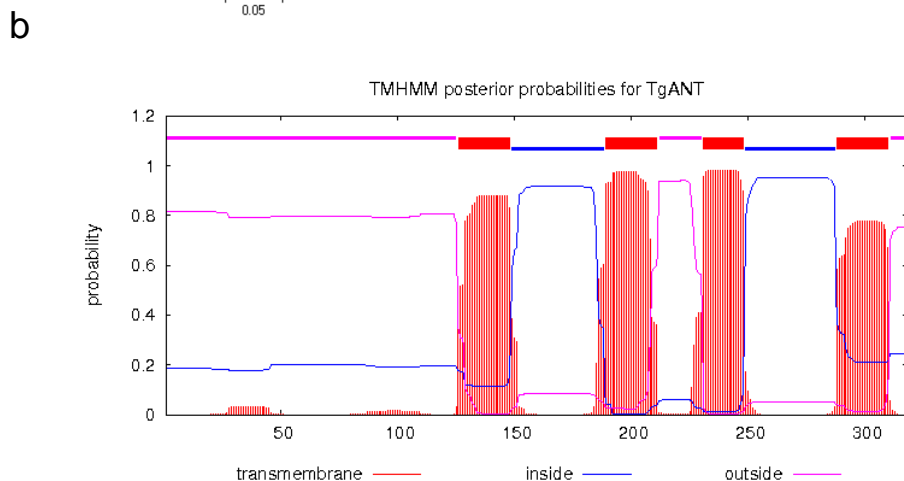

Supplement: Supplementary file 2 — Additional file 2: Figure S1. a protein sequence alignment of TgANT using MEGA7 from Toxoplasma gondii, Saccharomyces cerevisiae, Plasmodium falciparum, Cyclospora cayetanensis, Arabidopsis thaliana, Cardiosporidium cionae, Homo sapiens and other species. b Prediction results of TMHMM Server v.2.0 showed that TgANT has four transmembrane domains. [file 13071_2022_5295_MOESM2_ESM.pdf]

Figure S2

a

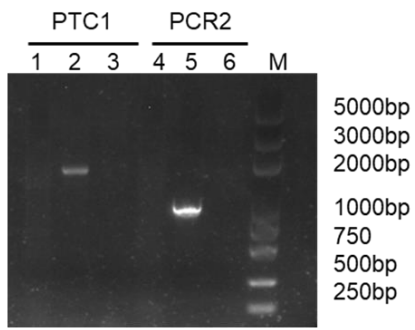

b

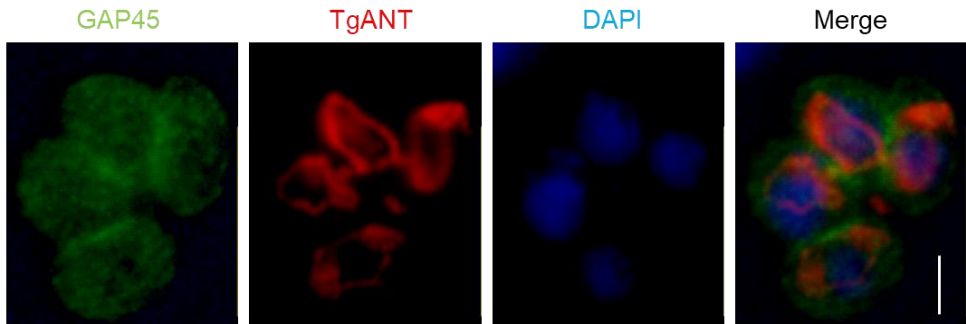

Supplement: Supplementary file 3 — Additional file 3: Figure S2. Identification of the iΔant gene complementation strain. a PCR1 and PCR2 were used to amplify the 5′ homologous recombination fragment (1890 bp) and the 3′ homologous recombination fragment (1218 bp), respectively. Lanes 2 and 5 are the experimental group; lanes 1 and 4 are the negative control group; lanes 3 and 6 are the blank control groups. b IFA assay. GAP45 (green) marks the outline of the parasite; red indicates the target protein TgANT; DPAI (blue) marks the nucleus. Scale bar: 2 μm. [file 13071_2022_5295_MOESM3_ESM.pdf]
